# Emerging alternatives to traditional anthelmintics: the in vitro antiparasitic activity of silver and selenium nanoparticles, and pomegranate (*Punica granatum*) peel extract against *Haemonchus contortus*

**DOI:** 10.1007/s11250-023-03722-0

**Published:** 2023-09-22

**Authors:** Ahmed M. Kaiaty, Fayez A. Salib, Sohila M. El-Gameel, Emil S. Abdel Massieh, Ahmed M. Hussien, Mohamed S. Kamel

**Affiliations:** 1 Department of Medicine and Infectious Diseases, Faculty of Veterinary Medicine, Cairo University, Giza, 11221 Egypt; 2 Parasitology Department, Faculty of Veterinary Medicine, Cairo University, Giza, 11221 Egypt; 3 Toxicology & Forensic Medicine Department, Faculty of Veterinary Medicine, Cairo University, Giza, 11221 Egypt; 4 General Organization for Veterinary Services, Giza, Egypt

**Keywords:** Barber’s pole worm, Parasiticidal effect, Plant medicine, Gastrointestinal parasites, Sheep parasites, Abomasum

## Abstract

*Haemonchus contortus* (*H. contortus*) is one of the most prevalent gastrointestinal nematodes, causing health problems and economic losses in ruminants. Nanotechnology holds great promise as a field of science, with potential applications in veterinary medicine. This study investigated the in vitro anthelmintic activity of silver nanoparticles (AgNPs), selenium nanoparticles (SeNPs), and pomegranate peel extract (*Punica granatum*; PPE) on different stages of *H. contortus*: eggs, larvae, and adults. The in vitro anthelmintic efficacy was evaluated using the egg hatching inhibition assay (EHA), the third larval stage paralysis assay (LPA), and the adult worm motility inhibition assay (WMI). Six dilutions of PPE were utilized for EHA, LPA, and WMI, ranging from 0.25 to 6 mg/ml. AgNPs dilutions ranged from 0.00001 to 1.0 μg/ml for EHA and LPA and 1 to 25 μg/ml for WMI. SeNPs were utilized at dilutions of 1, 5, 10, and 15 μg/ml for EHA, LPA, and WMI. The results showed that the lowest concentration of AgNPs, SeNPs, and PPE significantly inhibited egg hatching. To further assess larvicidal activity, AgNPs at the highest concentration of 1 μg/ml induced a strong larvicidal effect, as did SeNPs at the lowest concentration. On the contrary, PPE displayed a significant larvicidal effect at 1 mg/ml compared to the control. The percentage mortality of adult *H. contortus* was measured as follows (mortality (%) = the number of dead adult *H. contortus*/total number of adult *H. contortus* per test × 100). The death of the adult *H. contortus* was determined by the absence of motility. Adult *H. contortus* mortality percentage was also significantly affected by all three agents when compared to the control. The AgNPs, SeNPs, and PPE have effective antiparasitic activity on gastrointestinal parasitic nematodes. These results provide evidence of the excellent antiparasitic properties of AgNPs, SeNPs, and PPE, demonstrating their effectiveness in controlling eggs, larvae, and adult *H. contortus in vitro*.

## Introduction

 Helminthiasis is a major obstacle to the development of livestock production, causing enormous economic losses in grazing areas (Adduci et al. 2022; Bricarello et al. 2023; Flay et al. 2022; Sargison 2012). Heavy infection of livestock with nematodes is one of the main causes of increased mortality, reduced meat production, and low fertility rates**.** In tropical, subtropical, and temperate areas, *Haemonchus contortus* (*H. contortus*) is a common gastrointestinal nematode that causes severe damage to ruminants (Bricarello et al. 2023; Paraud and Chartier 2017).

The use of chemical anthelmintics to treat and control nematodes is common. However, these anthelmintics are expensive, and resistance to them has developed over time (Gilleard et al. 2021; Tinkler 2019; Vercruysse et al. 2018). While benzimidazoles are effective against roundworms and flatworms, they have limitations such as low solubility and short residence times (Lanusse et al. 2018; Paraud and Chartier 2017). Additionally, *H. contortus* has developed resistance to various anthelmintics, including benzimidazoles, albendazole, and imidazothiazoles (Ali et al. 2021b; Kotze and Prichard 2016). Reports of resistance to currently available drugs (e.g., albendazole, levamisole, and fenbendazole) have also been documented (Flávia da Silva et al. 2018).

In this context, plant-based alternatives have gained attention as environmentally safe and effective options for controlling internal nematodes (Eguale et al. 2007; Kamaraj et al. 2010; Kanojiya et al. 2014). *Punica granatum* L., commonly known as pomegranate, is a deciduous shrub or tree belonging to the genus Punica and the family Lythraceae. It is native to Iran and northern India but widely distributed in the Mediterranean region, growing from sea level to 800 m altitude (Castagna et al. 2021). Pomegranate has been extensively used in folk medicine for its remedial properties against various disease, including intestinal worms, inflammation, dysentery, persistent cough, and diarrhea (Ismail et al. 2012). The fruit, peels, seeds, and leaves of pomegranate contain bioactive compounds such as phenolic acid, anthocyanins, flavonoids, hydrolyzable tannins, and other polyphenols. These compounds interfere with energy production pathways in parasites, leading to paralysis and death (Veerakumari and Munuswamy 2000). A pomegranate-based compound has been recommended to reduce gastrointestinal nematode (GIN) egg production by 50%, aligning with the sustainable and complementary green veterinary pharmacology (GVP) approach that aims to reduce chemical use and counteract anthelmintic resistance (Castagna et al. 2021). Studies have demonstrated the effect of methanol extracts of pomegranate fruit peels on larval motility and the egg hatchability rate of *H. contortus* parasites (Jabeen et al. 2015, Da Silva Felix et al. 2022) (Ahmed et al. 2020; Castagna et al. 2020). However, challenges such as efficacy variability, potential resistance, limited availability, and potential antinutritional effects need to be addressed (Athanasiadou et al. 2007; Jamil et al. 2022; Valladão et al. 2015)*.*

Nanotechnology offers the potential to enhance the benefits of anthelmintics through improved regulation of drug delivery, targeted action, and reduced systemic dissemination and side effects (Bai et al. 2018). The cost-effectiveness and availability of nanomaterials make them ideal for veterinary applications (Bai et al. 2018; El-Sayed and Kamel 2018, 2019). Silver nanoparticles (AgNPs) prepared from the aqueous extract of *Azadirachta indica* have been analyzed for their anthelmintic activities against *H. contortus* (Tomar and Preet 2016). Selenium nanoparticles (SeNPs) have been found to exhibit anthelmintic effects on the protoscoleces of *Echinococcus granulosus* (*E. granulosus*) and prophylactic effects on acute toxoplasmosis (Mahmoudvand et al. 2014; Shakibaie et al. 2020). A recent systematic meta-analysis concluded that nanoparticles could be potential therapeutic agents as novel anthelmintics for controlling *H. contortus* (Ali et al. 2021a). Therefore, this study aims to investigate the anthelmintic activities of *Punica granatum* L. and nanoparticles, particularly silver and selenium nanoparticles, against *H. contortus* in vitro.

## Materials and methods

### Synthesis of silver nanoparticles

**Fig. 1:**
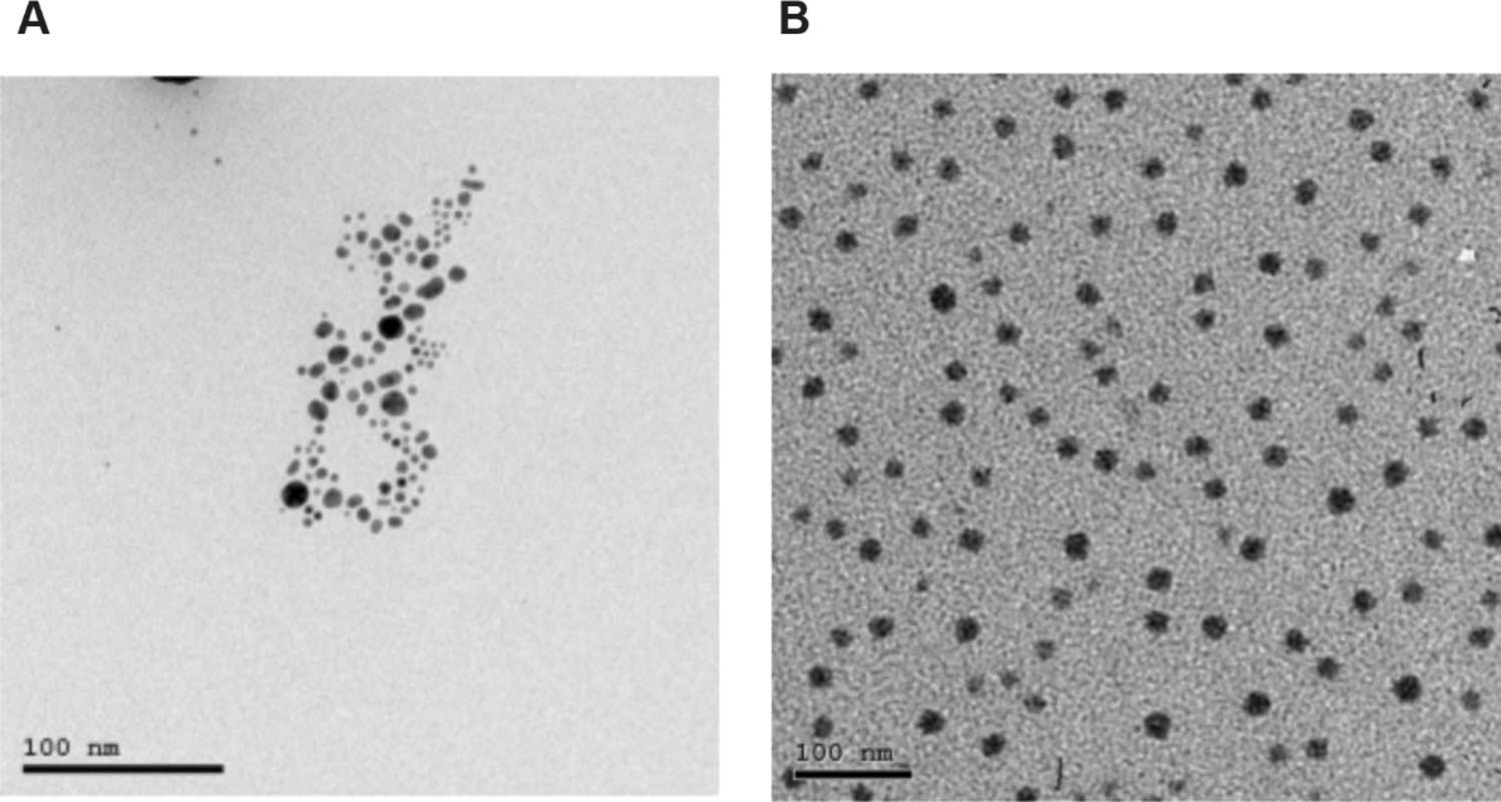
High-resolution TEM showing the shape and size of AgNPs (**A**) and SeNPs (**B**). These images were acquired and obtained from NanoTech Egypt

AgNPs were acquired from NanoTech Egypt, a photo-electronics company (Fig. 1). The AgNPs were synthesized via a chemical reduction method adapted from a protocol by El Mahdy et al. (2015). The process started by solubilizing 1.89 g NaBH4 into 50 ml of distilled water, which was then cooled. 0.272g AgNO_3_ was added to 345 ml of distilled water, and the combination was stirred for 15 min with a magnetic stirrer. Subsequently, 0.504g of polyvinylpyrrolidone (PVP) (CDH®) and 2.912g of 205 trisodium citrate (ADWIC®) were dissolved in 48 ml of distilled water, mixed for 15 min, and then added to the AgNO_3_ solution. Lastly, 8 ml of cold-reducing NaBH4 solution was added to the mixture and stirred vigorously for 30 min. The reduction reaction was completed when the solution became dark yellow or brown in color. The stock solution was stored at 4°C away from direct light in a black-colored bottle. Subsequently, transmission electron microscopy (TEM), enabled by a JEOL JEM2100, was executed at a 200-kV accelerating voltage. Additionally, the UV-Vis absorption spectra were precisely determined by an Ocean Optics USB 2000 + VISNIR fiber optical spectrophotometer. This commercially supplied AgNPs produced by NanoTech Egypt was previously characterized in several publications using spectrophotometric assays, transmission electron microscopy, FTIR, and XRD (Abou Elez et al. 2021; El-Ashram et al. 2020; Fouad et al. 2021; Ouf et al. 2017). The AgNPs exhibited a spherical shape with an average diameter of 20 ± 5 nm and it was dispersed in deionized water (DW) (Abou Elez et al. 2021; El-Ashram et al. 2020; Fouad et al. 2021; Ouf et al. 2017). The absorption spectrum of the AgNPs displayed a peak at 410 nm (Abou Elez et al. 2021; El-Ashram et al. 2020; Fouad et al. 2021; Ouf et al. 2017). The Ag-NPs were found to exist as either individual spherical particles or as aggregated clusters (Abou Elez et al. 2021; El-Ashram et al. 2020; Fouad et al. 2021; Ouf et al. 2017).

### Synthesis of selenium nanoparticles

The synthesis of SeNPs was purchased from NanoTech Egypt for a photo-electronics company (Fig. 1). According to Vahdati and Tohidi Moghadam (2020), SeNPs were prepared by reducing sodium selenite with ascorbic acid, and then stabilized with polysorbate 20. The procedure entailed the addition of 30 mg Na2SeO3.5H2O to 90 ml of Milli-Q water and, with vigorous stirring, ascorbic acid (10 ml, 56.7 mM) was then applied dropwise. After each 2 ml of ascorbic acid, 10 μl of polysorbate were incorporated. This reaction caused the transition of the clear white reactant solution to a clear red color, signifying the formation of SeNPs. All operations were performed in an aseptic environment to ensure sterility using only Milli-Q water and a sterile cabinet. The resultant sample was then centrifuged at 12,000 rpm to obtain the SeNPs, which could later be re-suspended in sterile double distilled water. An inductively coupled plasma optical emission spectrophotometer (ICP-OES, model Vista-Pro from Varian) was utilized to determine the selenium quantity of the nanoparticles. Subsequently, TEM, enabled by a JEOL JEM2100, was executed at a 200-kV accelerating voltage. The production of nanoselenium was completed via the oxidation reaction method. The SeNPs provided by NanoTech Egypt have undergone analysis, utilizing spectrophotometric assays, transmission electron microscopy (El-Borady et al. 2020). The SeNPs displayed a semispherical shape, with an average diameter of 45 nm (El-Borady et al. 2020).

### Pomegranate peel extract

#### Preparation of pomegranate extract (PPE)

Pomegranate nuts were purchased and washed with water during cleaning and processing to remove foreign bodies such as leaves, soil, stems, and gravel. The skin was removed from the flesh, and the pomegranate seeds were crushed using a Wiley mill, Swedesboro, NJ, USA. Then, the pomegranate peels were dried in the sun until they were completely dry before being used. The pomegranate peel methanol extract was beaten at a ratio of 1:2:2 (w peel/v water/v methanol) and stored at 4 °C/48 h. The pomegranate peel methanol extract was then used as a solvent. The extract was then sieved, pressed at 40–50 °C to remove the solvent, stored at 3–4 °C, and labeled as pomegranate methanol extract (Abdel Moneim 2012). Then, the extract receives a 100% dose (100% PPE = 500 g + 500 ml of water) (Amelia et al. 2017).

### Albendazole 2.5%

A 100 ml solution of 2.5g of albendazole (2.5% w/v) (manufactured by Pharma Swede of Egypt) was used as a positive control in the experiment.

### Sample collection

Sample collection was performed according to the World Association for the Advancement of Veterinary Parasitology (WAAVP) guidelines (Tomar and Preet 2016). The adult *H. contortus* worms, both females and males, were collected directly from the abomasum of sheep naturally infected with *H. contortus* and were slaughtered at the Al-Basatin abattoir in Cairo, Egypt. The samples were then placed into labeled and sterilized screw-capped test tubes in an icebox and transferred to the laboratories of Cairo University’s Faculty of Veterinary Medicine. The collected females were placed in phosphate-buffered saline (PBS, 7.2 pH), finely chopped to extract the eggs, and washed with distilled water. Then, the collected eggs were then mixed with fecal samples from sheep that had been previously tested and found to be free of any parasitic infection. This fecal sample was used as a suitable medium for egg culture to further collect larvae for our experiment.

### Egg counting technique

The number of eggs was determined using the McMaster technique as described by Kates and Soulsby (1969). Two grams of fecal samples was mixed with 58 ml of saturated sodium chloride solution and then sieved. A pipette of the collected suspension was immediately placed in the compartment of the McMaster Slide control chamber. The number of eggs in both chambers was counted after 10 min under a light microscope (Labomed®). The following formula was used to determine the total number of eggs detected: Number of eggs per gram (count) (epg) = ((total number of eggs counted in both chambers/number of counting chambers) × 100).

### Coproculture

For in vitro propagation, coprocultures were performed according to Pilo et al. (2012). Briefly, a combination of 40 g of feces and collected eggs is mixed with sawdust, sealed in a jar, and maintained at a temperature of approximately 26 °C for 14 days with daily moistening and aeration. Subsequently, the larvae were separated via the Baermann method and identified according to the morphological keys proposed by Van Wyk and Mayhew (2013). After that, the collected L3 were stored in a tube containing water at 4 °C (Van Wyk and Mayhew 2013).

### Egg hatchability assay

According to the methods of Davuluri et al. (2019), the in vitro effects of AgNPs, SeNPs, and PPE on egg hatchability were studied. AgNPs, SeNPs, and PPE were used as the test treatments, albendazole served as the positive control, and untreated eggs in PBS were used as the negative control (Davuluri et al. 2019). PPE was used at concentrations of 0.25 mg, 0.5 mg, 1 mg, 2 mg, 4 mg, and 6 mg in 1 ml PBS (Amelia et al. 2017; Castagna et al. 2020). AgNPs were used at concentrations of 0.00001, 0.0001, 0.001, 0.01, 0.1, and 1 μg/ml (Tomar and Preet 2016). Selenium nanoparticles (SeNPs) were used at concentrations of 1, 5, 10, and 15 μg/ml (Mahmoudvand et al. 2014). Then, 0.5 ml of each concentration was added to 0.5 ml of water containing 400 eggs. For the positive control, 0.5 ml of each concentration of albendazole (40, 20, 10, 5, 2.5 μg/ml PBS) was added to 0.5 ml of water containing 400 eggs. The eggs in PBS without extract or drugs were used as a negative control. After the test tubes were covered and incubated at 27°C for 48 h, Lugol’s iodine solution was dropped into each test tube to stop egg hatching. Then, all the supernatant was removed, and the sediment was examined with an anatomical microscope (Labomed®) to determine the number of hatched larvae. The percent inhibition = (number of hatched larvae in the negative control − number of L1 in the other test groups/number in the negative group) × 100 (Haque et al. 2014).

### Larval inhibition test

As per Hounzangbe-Adote et al. (2005), the effect of AgNPs, SeNPs, and PPE on larval inhibition was studied in vitro. Fifty L3 were added to 0.5 ml of each concentration of PPE (0.25 mg, 0.5 mg, 1 mg, 2 mg, 4 mg, and 6 mg in 1 ml PBS) (Amelia et al. 2017; Castagna et al. 2020), AgNPs (0.00001, 0.0001, 0.001, 0.01, 0.1, and 1 μg/ml) (Tomar and Preet 2016), and SeNPs (1, 5, 10, and 15 μg/ml) (Mahmoudvand et al. 2014). For the positive control, 50 L3 were added to 1 ml of water mixed with 1 ml (30 μg albendazole/ml PBS). For negative control, 50 L3 was used in PBS without extract or drugs. All tubes were incubated at 27°C for 24 h and then examined to determine the number of live, dead, or paralyzed larvae. The numbers of immobile (dead) and motile (live) larvae were counted under an anatomical microscope and recorded for each concentration. Mortality rate (M) was determined for each concentration according to Tomar and Preet (2016) (mortality (%) = (number of dead larvae/total number of larvae per test) ×100).

### Worm motility inhibition

Per Hounzangbe-Adote et al. (2005), the effect of AgNPs, SeNPs, and PPE on adult *H. contortus* was demonstrated in vitro. The abomasum of freshly slaughtered sheep was used to obtain the adults. Directly post-slaughter, the abomasums of the animals were collected and sent to the lab. Collected parasites were thoroughly washed using normal saline prior to their placement in PBS. Ten active-moving adult worms were added to each of the continuous concentrations of PPE (0.25 mg, 0.5 mg, 1 mg, 2 mg, 4 mg, and 6 mg in 1 ml of PBS ) (Amelia et al. 2017; Castagna et al. 2020), AgNPs (1, 5, 10, 15, 20, and 25 μg/ml of water) (Tomar and Preet 2016), and SeNPs (1, 5, 10, and 15 μg/ml) (Mahmoudvand et al. 2014). On the other hand, the negative control is PBS with only ten worms in PBS, while the positive control is ten worms in 30 μg albendazole dissolved in DMSO and diluted in ml PBS. All tubes were incubated at 28°C. Subsequently, adult motility was measured every 4 h for 12 h. The numbers of immobile (dead) and motile (live) worms were counted and recorded for each concentration. The mortality % was determined for each concentration according to Tomar and Preet (2016). Mortality (%) = the number of dead adult stage/total number of adult stage per test × 100.

### Statistical analysis

Statistical analysis was conducted utilizing SPSS 25 software. Chi-square and Fisher’s exact test with Yates correction for continuity were adopted to analyze the data. Nonlinear regression was implemented after data normalization to determine LD50. Data were analyzed using nonlinear regression to calculate LD50 values, which indicate the concentration of the substance that causes 50% mortality (Aggarwal et al. 2022; Matsumoto et al. 2021; Yim et al. 2021). A *p*-value under 0.05 was considered statistically significant.

## Results

### The anthelmintic activity of AgNPs, SeNPs, and PPE on *H. contortus* eggs, larvae, and adults

**Fig. 2:**
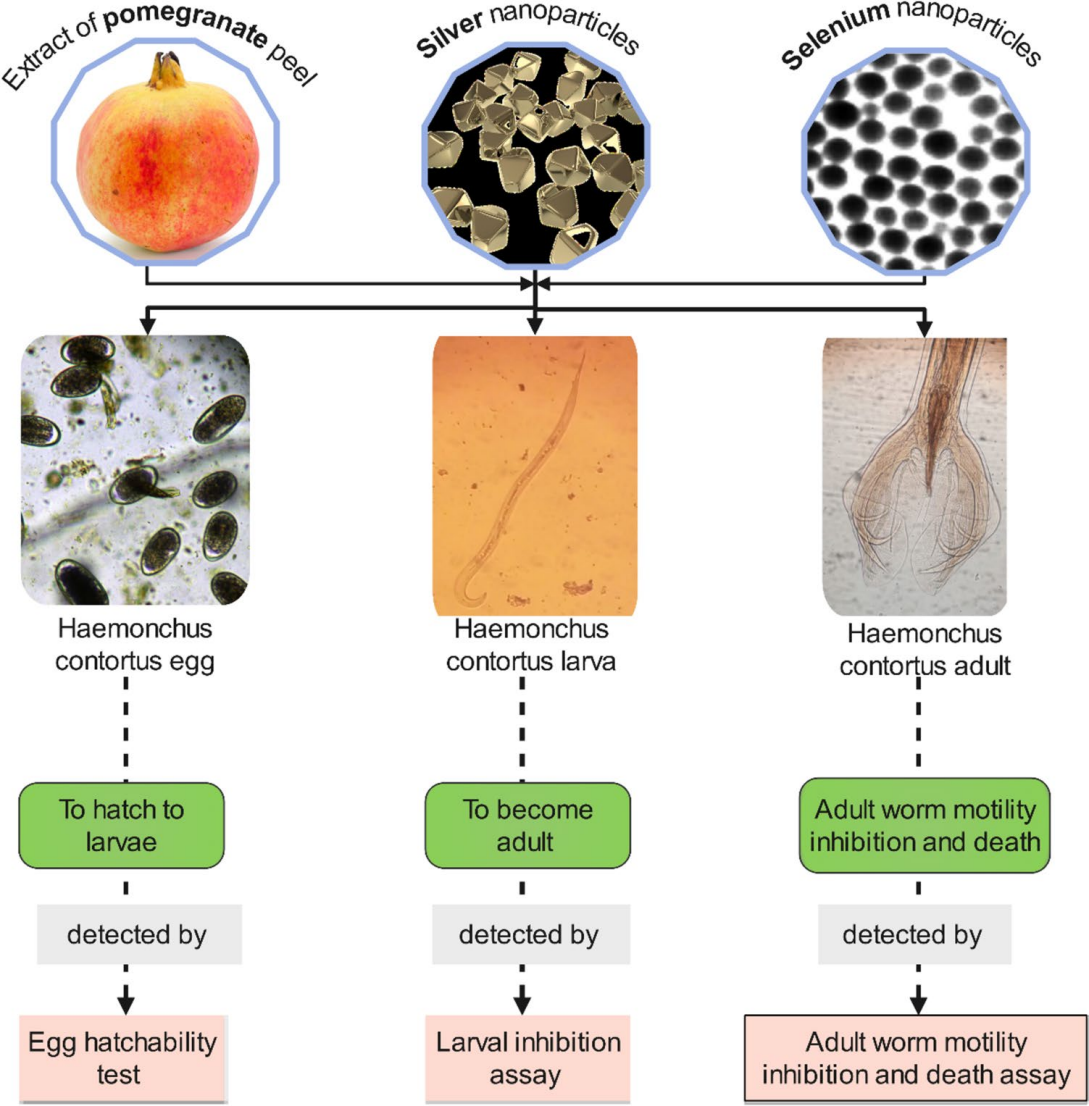
The in vitro assessment of the three extracts (silver nanoparticles, selenium nanoparticles, and pomegranate peel extract) on *H. contortus* nematode eggs, larvae, and adults

**Fig. 3:**
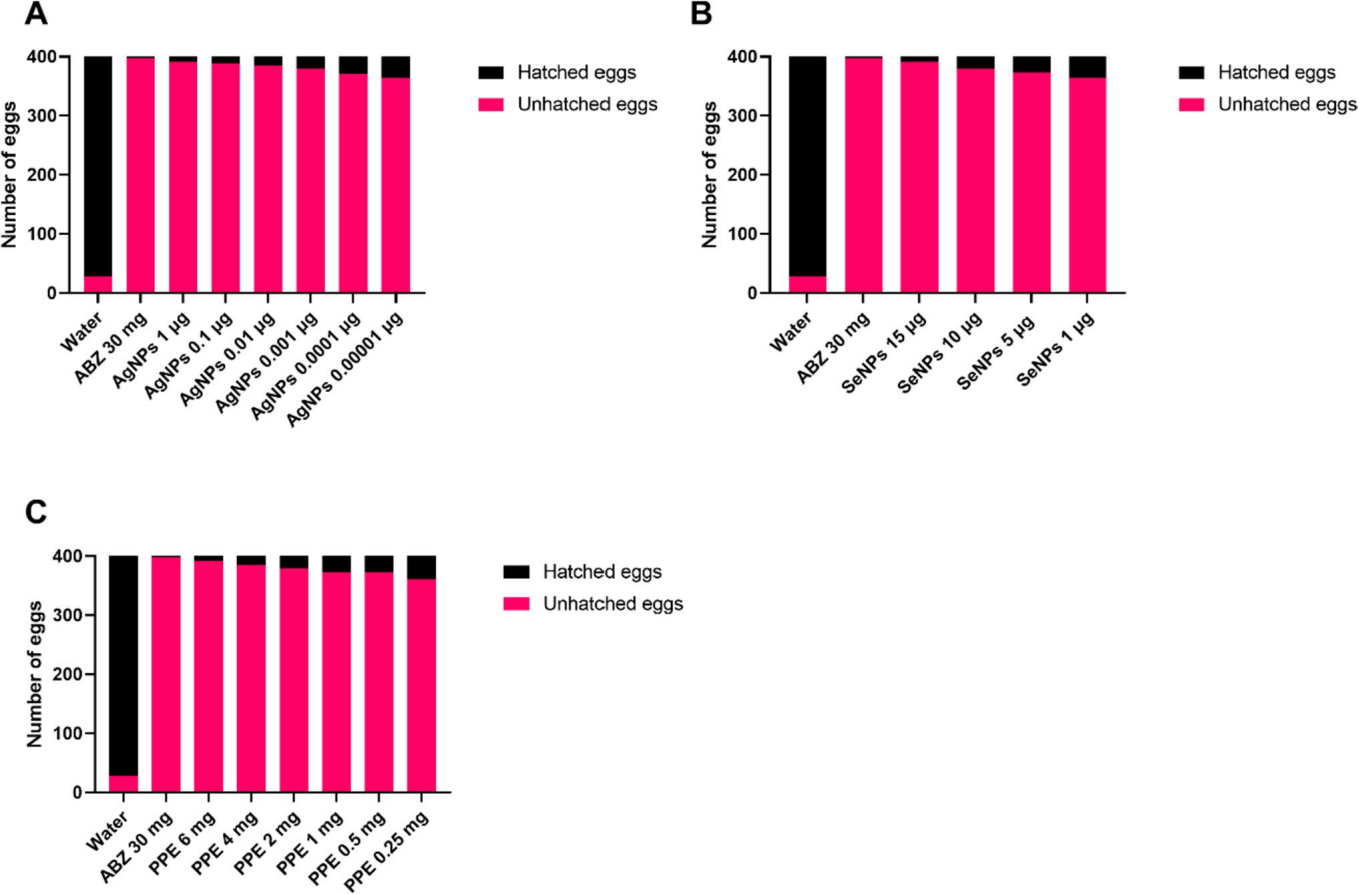
The effect of different concentrations of AgNPs (**A**), SeNPs (**B**), and PPE (**C**). on the egg hatching of *H. contortus*. All the concentrations of the three compounds have a significant effect on the egg hatchability using the chi-square test. A *P*-value <0.05 is deemed significant

**Fig. 4:**
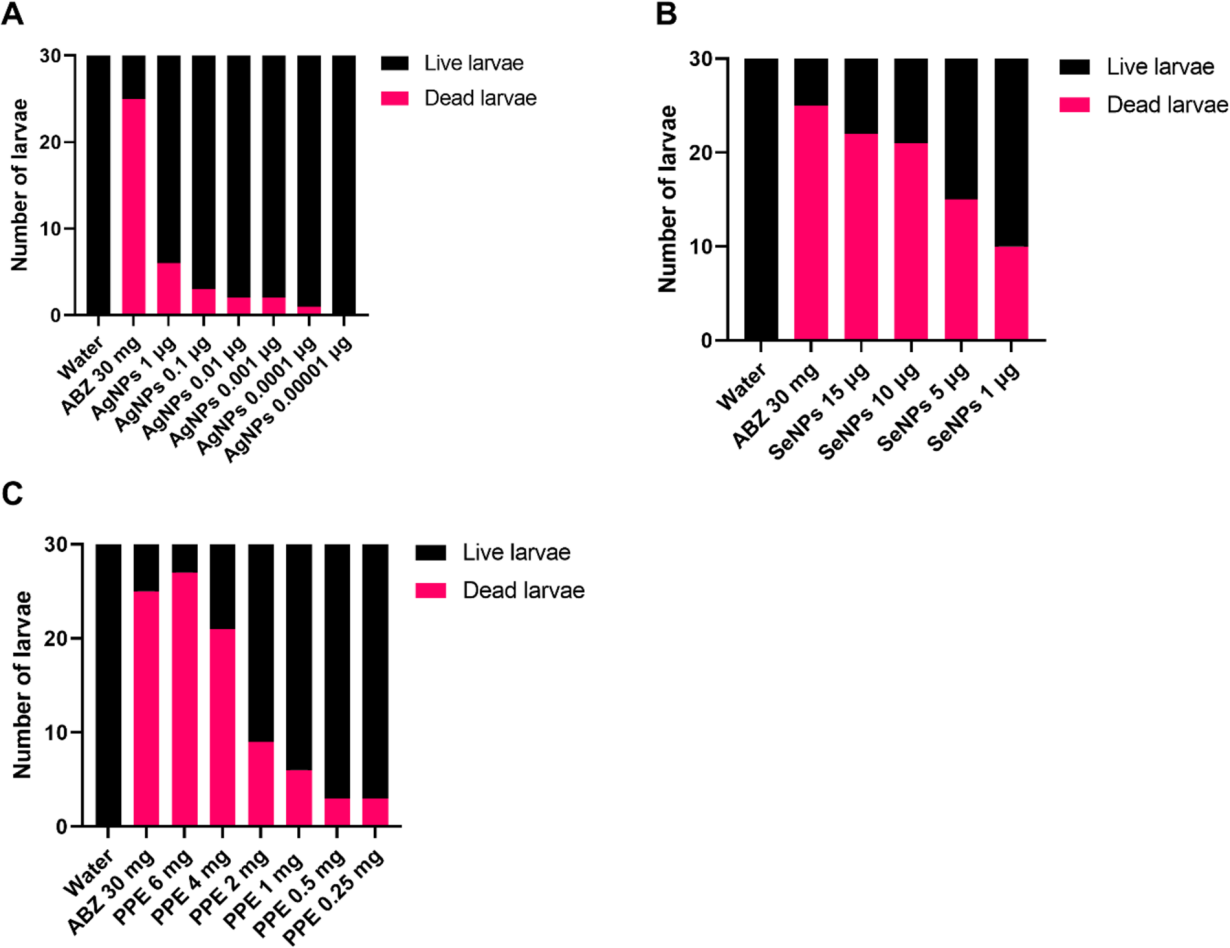
The effect of different concentrations of AgNPs (**A**), SeNPs (**B**), and PPE (**C**) on inhibiting the larvae of *H. contortus*. All the concentrations of the three compounds have a significant effect on the larva using the chi-square test, starting from 1 μg for AgNPs, 1 μg for SeNPs, and 1 mg for PPE and increasing the significance with increased concentration. A *P*-value <0.05 is deemed significant

**Fig. 5:**
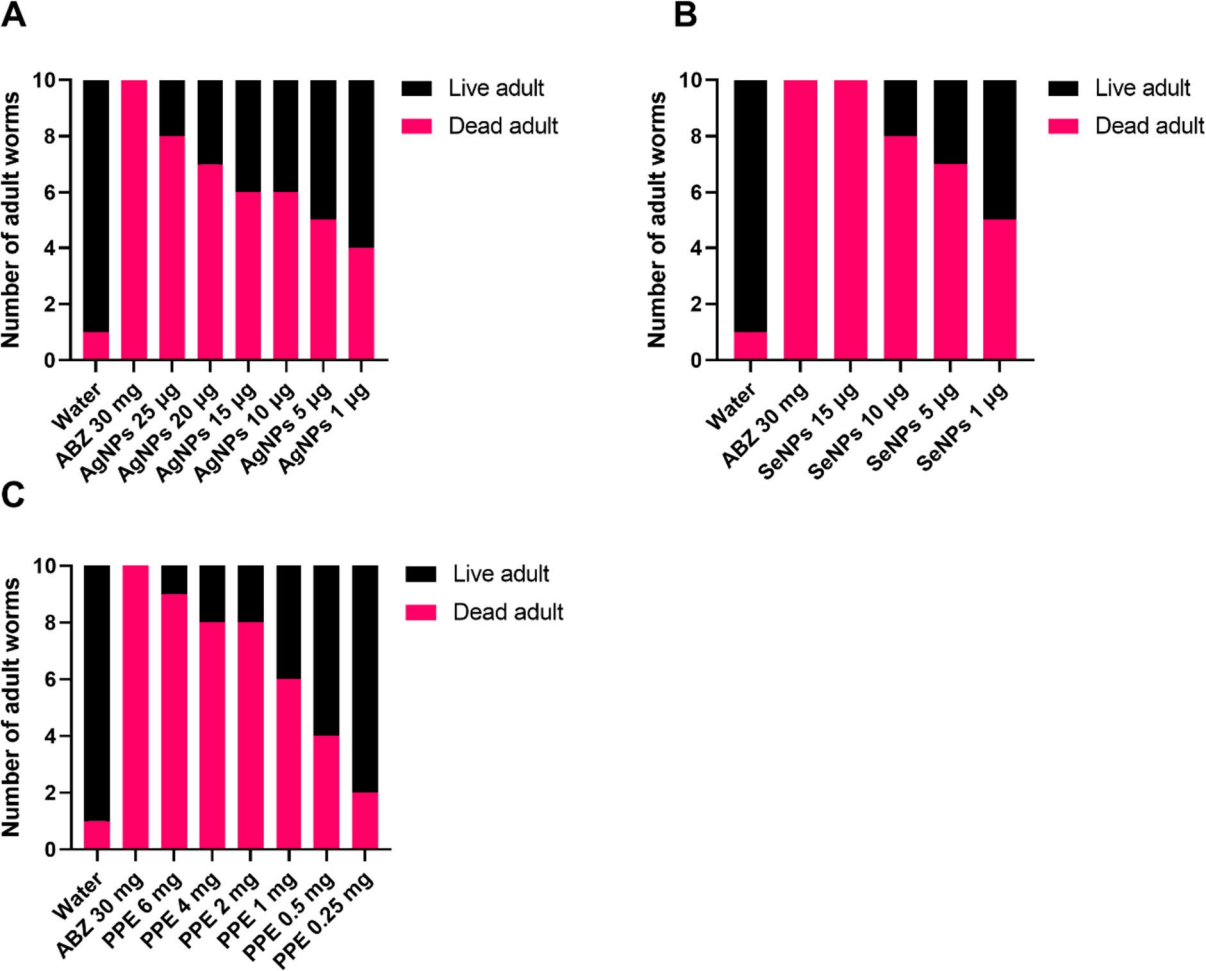
The effect of different concentrations of AgNPs (**A**), SeNPs (**B**), and PPE (**C**) on the adult *H. contortus* exposed to the three compounds showed a significant effect using the chi-square test, starting from 10 μg for AgNPs, 5 μg for SeNPs, and 1 mg for PPE and increasing the significance with increased concentration. A *P*-value <0.05 is deemed significant

Figure 2 shows the schematic in vitro assessment approach of AgNPs, SeNPs, and PPE on *H. contortus* nematode eggs, larvae, and adults. The inhibition of egg hatching caused by AgNPs at 48 h exhibited a high degree of significance at the lowest concentrations of 0.00001 μg/ml as compared to control (*p*-value<0.0001), while the larvicidal activities at 24 h and adulticidal activities were found to be significant at concentrations of 1 μg/ml (*p*-value; 0.0237) and 10 μg/ml (*p*-value; 0.0191) and above, respectively. Thus, at a concentration of 1 μg/ml, AgNPs significantly showed ovicidal (*p*-value<0.0001) and larvicidal (*p*-value; 0.0237) effects against *H. contortus* but not an adulticidal effect (*p*-value; 0.1213), while at 10 μg/ml, it significantly affected the eggs, larvae, and adults (Figs. 3, 4, and 5).

In the case of SeNPs, EHA, LPA, and WMI were significantly achieved at concentrations of 1 and 5 μg/ml and above compared to the control groups. The concentration of 5 μg/ml showed a significant effect on all *H. contortus* stages, while at a concentration of 1 μg/ml, it affected only the eggs and larvae with *p*-value <0.0001 and *p*-value =0.0008, respectively (Figs. 3, 4, and 5).

The lowest concentrations of PPE that showed inhibition of EHA, LPA, and WMI were 0.25, 1, and 1 mg/ml showing *p*-value <0.0001, *p*-value=0.0098, and *p*-value=0.0191, respectively. The 1 mg/ml concentration showed significant ovicidal, larvicidal, and adulticidal activity against *H. contortus*, while the 0.25 mg/ml showed significant activity against the eggs only (*p*-value <0.0001) (Figs. 3, 4, and 5).

### Lethal dose (LD50)

**Fig. 6:**
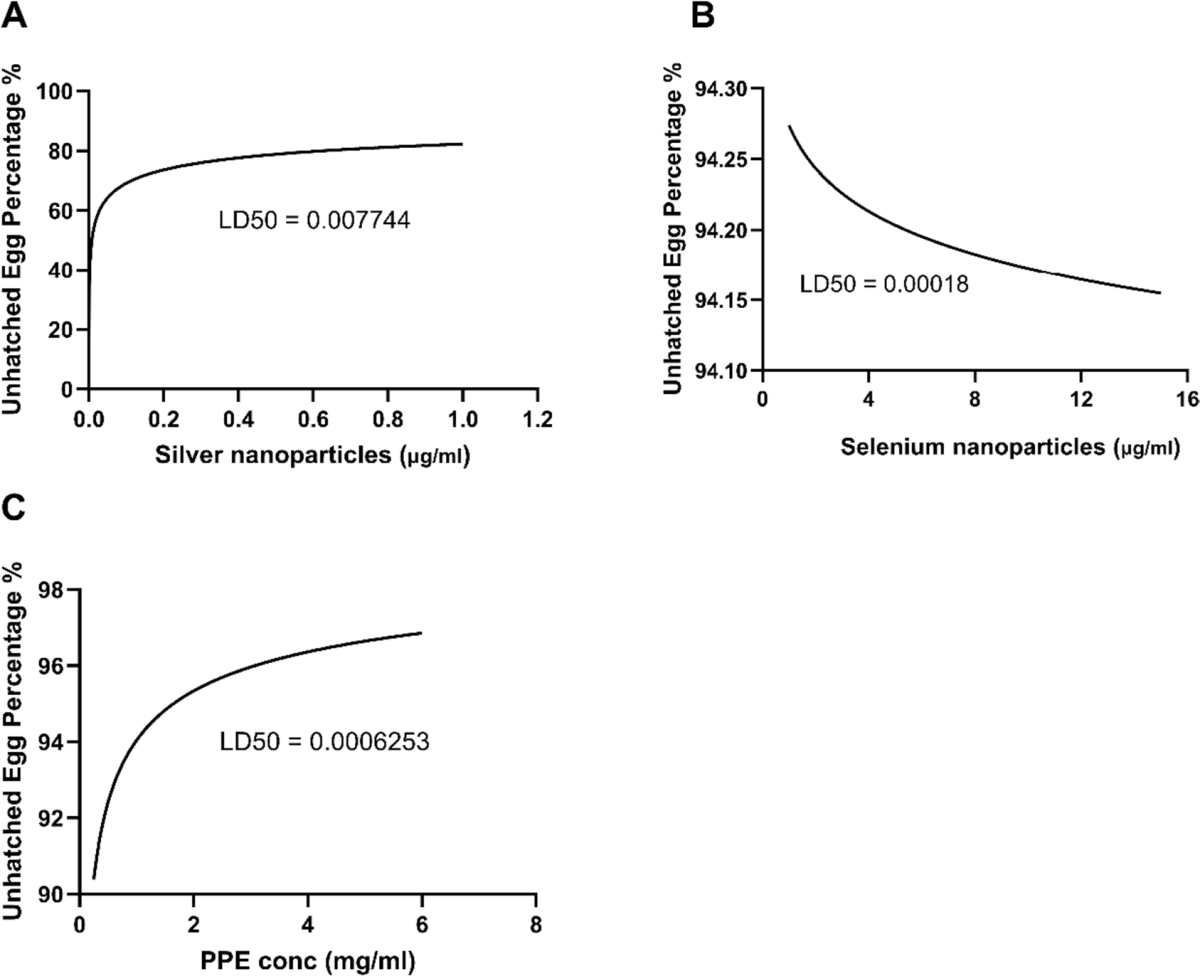
The LD50 of AgNPs (**A**), SeNPs (**B**), and PPE (**C**) required for affecting the *H. contortus* egg hatchability

**Fig. 7:**
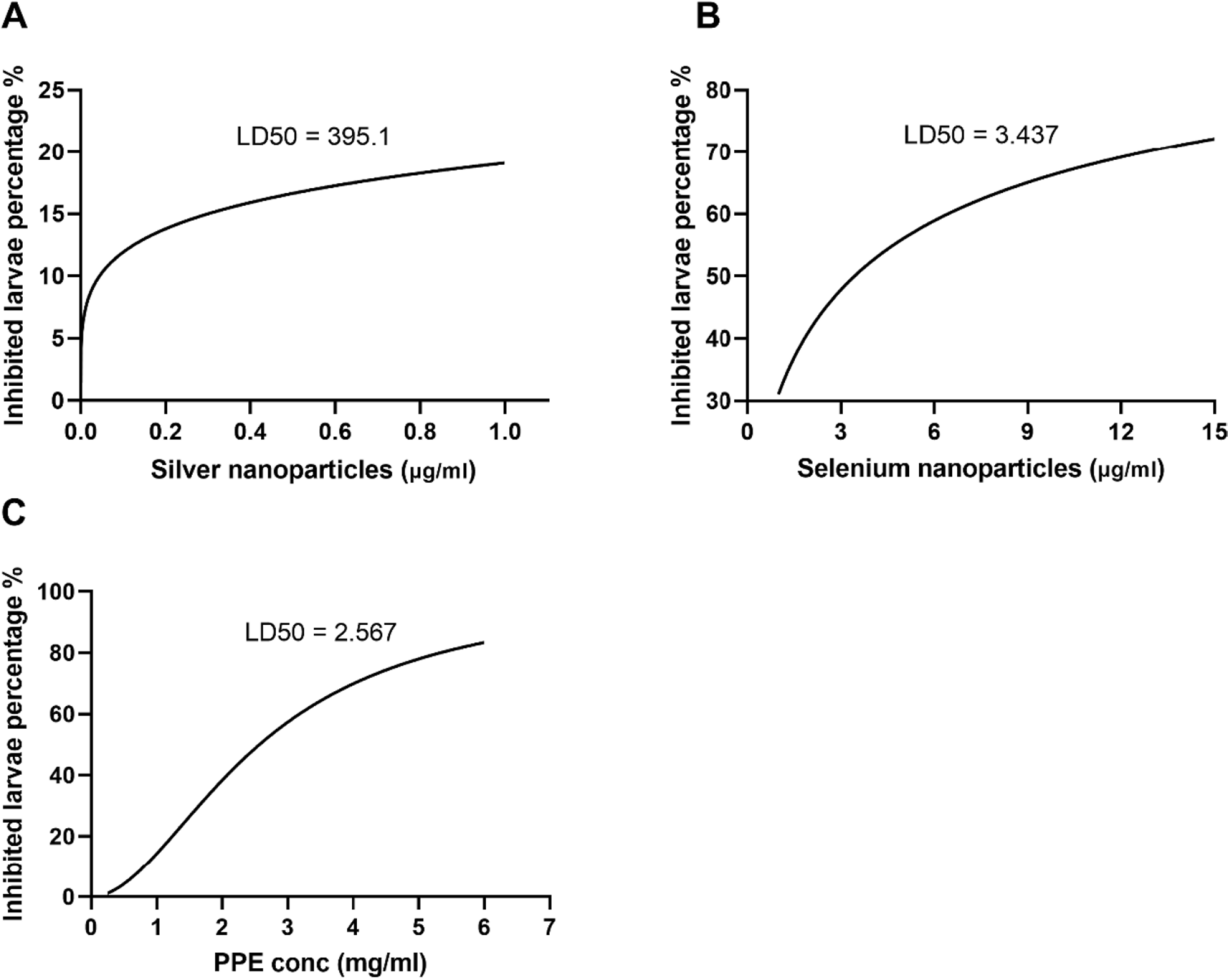
The LD50 of AgNPs (**A**), SeNPs (**B**), and PPE (**C**) against *H. contortus* larvae

**Fig. 8:**
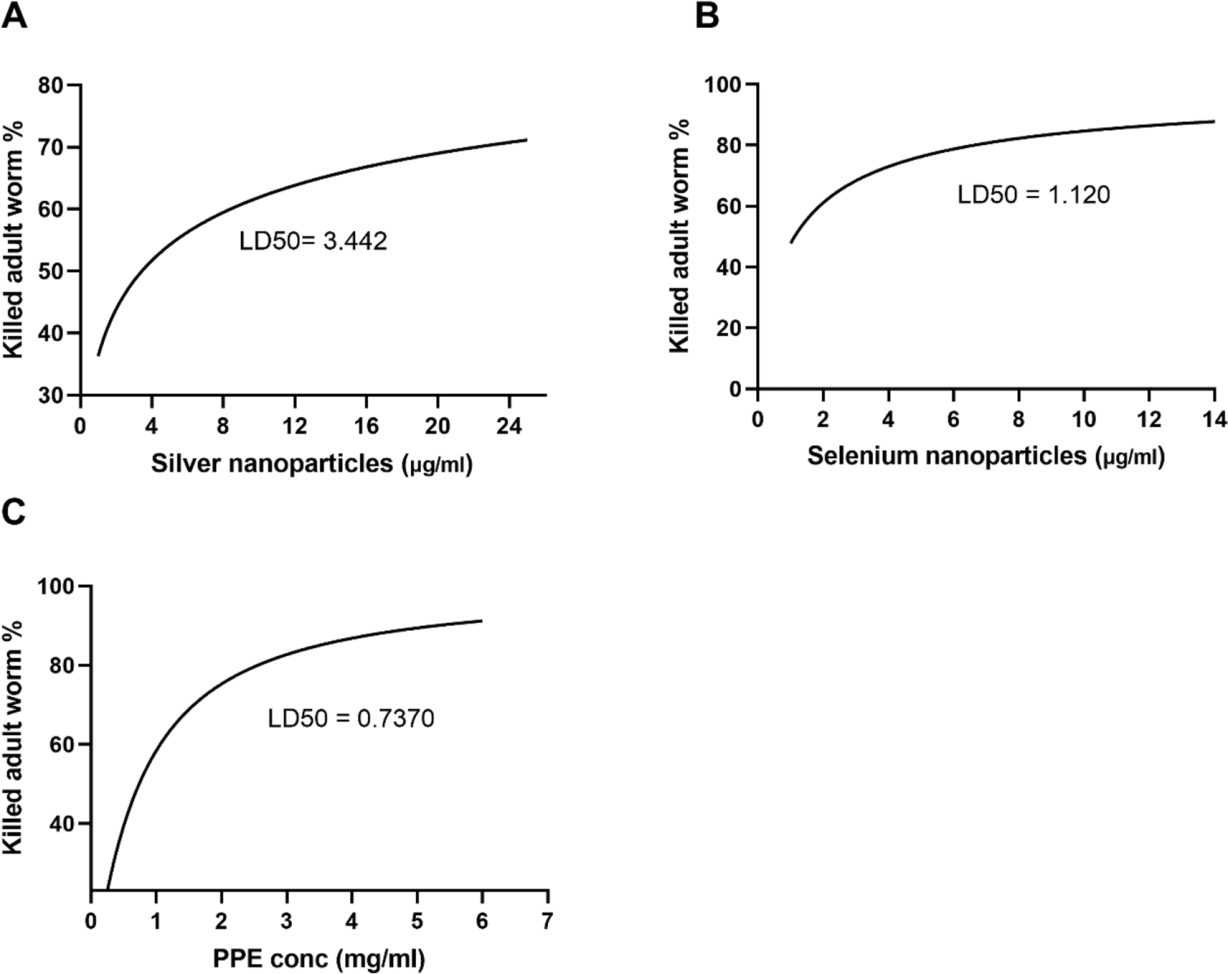
LD50 of AgNPs (**A**), SeNPs (**B**), and PPE (**C**) against *H. contortus* adult worms

Mortality data from *H. contortus* eggs, larvae, and adults receiving graded doses of AgNPs, SeNPs, and PPE were recorded after exposure, and the LD50 is calculated and shown in Figs. 6, 7, and 8. Based on the LD50 shown in the figures, we can determine the dose that affects/kills 50% of any *H. contortus* stage (eggs, larvae, and adults).

## Discussion

*H. contortus* is a notable helminth in ruminants that grows in the abomasum and feeds upon their blood, leading to a wide range of signs and mortality in the most serious situations. Global production losses due to anemia, body weight decrease, and even death have been noted (Sargison 2012). Nowadays, it is imperative to design innovative antiparasitic drugs because of the advent of many resistances among internal parasites (Zajíčková et al. 2020). Plant-based anthelmintics and nanotechnology approaches may be promising alternatives to synthetic anthelmintics in order to combat parasitic gastroenteritis infections (Akhtar et al. 2000). The use of nanoparticles as potential new anthelmintic candidates is attributed to their small size and high surface reactivity, both of which offer considerable potential for biomedical applications (Adeyemi and Whiteley 2013). Additionally, nanoparticles can penetrate membranes, generating reactive oxygen species (ROS), which are known to effectively kill parasites (Ali et al. 2021b; Bhardwaj et al. 2012). Therefore, investigations into the anthelmintic activity of nanoparticles have recently increased, with the primary aim of evaluating their potential to control parasitic infections (Esmaeilnejad et al. 2018).

The current study provided evidence for the highly significant anthelmintic activity of AgNPs, SeNPs, and PPE in various concentrations against *H. contortus*. AgNPs exhibited significant suppression of egg hatching at an extremely low concentration of 0.00001 μg/ml, with larvicidal effects observed at 1 μg/ml and adulticidal impacts at concentrations of 10 μg/ml and higher. Similarly, SeNPs demonstrated significant efficacy against egg hatching, larvae, and adult stages at concentrations of 1 μg/ml and above. PPE displayed strong effects at concentrations of 0.25, 1, and 1 mg/ml, with a remarkable concentration of 1 mg/ml exhibiting ovicidal, larvicidal, and adulticidal activity against *H. contortus*, while the concentration of 0.25 mg/ml slowly affected the eggs. In the same context, Costa et al. (2008) discovered a more effective inhibition of *H. contortus* egg hatchability utilizing the ethanol extract of *Azadirachta indica* over ethyl acetate extract, which attained a 99.77% inhibitory rate at a concentration of 3.12 mg/ml. Moreover, the efficacy of the PPE was investigated in vivo with GINs in sheep (Kaiaty et al. 2021), detecting a 97% decrease in eggs in feces by the 21st day. Previous trials by da Silva Felix et al. (2022) ascertained the ovicidal effect of *P. granatum* peel salt extract, which held key secondary metabolites and revealed non-toxic responses at the lowest concentration tested. In addition, Kaiaty et al. (2021) found that PPE presented anthelmintic activities in various ruminant species, such as cattle, buffalo, sheep, and goats, when compared to synthetic anthelmintics. Furthermore, Anjos et al. (2016) also reported a larvicidal effect of PPE against *H. contortus* in goats and cattle. It is presumed that the tannins found in PPE are the most oxidative element, obstructing phosphorylation and ATP synthesis in *H. contortus* (Martin 1997). Furthermore, the antiparasitic effects of PPE against *H. contortus* and other parasites are inferred to be the result of tannin and flavonoid molecules contained within components (Akkari et al. 2008; Barrau et al. 2005; El-Sayed et al. 2021; Hoste et al. 2006). Results of preceding studies substantiate the anthelmintic action of PPE against *H. contortus* in sheep in vivo and in vitro (Castagna et al. 2020, 2021, 2022; Hassan et al. 2020; Poli et al. 2021).

Our results demonstrated that AgNPs, SeNPs, and PPE have potent anthelmintic effects against adult *H. contortus* worms, with a significant rise in mortality being observed after 12 h when compared to control groups. Furthermore, the data demonstrated an increased mortality rate in *H. contortus* larvae when exposed to AgNPs, SeNPs, and PPE versus the control group. The results of AgNPs regarding anthelmintic activity against the *H. contortus* worm align with the conclusions of Tomar and Preet (2016) and Barbosa et al. (2019), who observed potent properties in a range of 1–25 μg/ml.

While the exact mechanism of action of AgNPs and SeNPs, against nematodes, is not yet completely understood, there is some experimental evidence to suggest how these nanoparticles may exert their toxic effects. AgNPs are known to have strong antimicrobial and antifungal properties, and studies have shown that they can disrupt cellular processes and metabolic pathways in a wide range of microorganisms and fungi (Anees Ahmad et al. 2020; Bruna et al. 2021; de Lacerda Coriolano et al. 2021; Nadhe et al. 2019). Similarly, SeNPs have been shown to have antifungal and antiviral effects, suggesting that they, too, may interfere with essential cellular processes (Ferro et al. 2021; Sharmin et al. 2021; Toprakcioglu et al. 2023). In addition, both AgNPs and SeNPs have been shown to induce oxidative stress and increase the production of reactive oxygen species, which can trigger cell death pathways (Afifi and Oshiba 2018; Ferro et al. 2021; Gad et al. 2021; KD and Venugopal 2022; Lin et al. 2021; Martínez-Esquivias et al. 2021; Rashidi et al. 2022; Vahdati and Tohidi Moghadam 2020). In general, the mechanism of nanoparticles may include disruption of the parasite cell membrane, inhibition of enzymatic activities, generation of ROS, induction of apoptosis, disruption of parasite metabolism, alteration of parasite gene expression, modulation of the immune system, physical damage, disruption of the reproductive system, disruption of the nervous system, and so on. Further research is needed to fully understand the mechanisms of action of these nanoparticles against nematodes. Goel et al. (2020) demonstrated that AgNPs are biocompatible, stable, and environmentally friendly when administered to worms and are associated with ROS- and NOS-dependent stress within 3 h. The anthelmintic activity of SeNPs against various parasites has been noted in prior research, including *murine Trichinella*, *Echinococcus granulosus*, and *Schistosoma mansoni*, corroborating our findings regarding *H. contortus* (Afifi and Oshiba 2018; Hikal et al. 2021; Pal and Zaheer 2022; Sarhan et al. 2022).

There is insufficient knowledge about the toxicological effects of selenium nanoparticles (SeNPs) in animals. In sheep, an oral dose of 1 mg/kg Se bw/day of SeNPs (smaller than 220 nm) or selenite showed similar toxicity (Kojouri et al. 2012a; Kojouri et al. 2012b). However, SeNPs were found to be less toxic and more bioactive than selenite in sheep (Bano et al. 2022). Some studies have evaluated the effect of sub-lethal doses of SeNPs on the health status of rats and found that sub-lethal doses of SeNPs did not cause any significant adverse effects on the rats (Qamar et al. 2020; Urbankova et al. 2021). The toxicological effects of SeNPs in animals are generally characterized by weight loss and increased mortality rate (Bano et al. 2022).

Our study provides evidence of the in vitro nematocidal effect of SeNPs, AgNPs, and PPE on ova, larva, and adult stages of *H. contortus.* This suggests that these nanoparticles have the potential as alternative measures against *H. contortus.* Further research is needed to determine their efficacy in vivo and in field trials. The use of nanoparticles as alternative measures against *H. contortus* is a relevant topic because of the increasing prevalence of anthelmintic resistance and the need for sustainable parasite control strategies. The lower toxic potency of SeNPs than dissolved ionic Se species suggests that SeNPs may be a safer alternative to traditional anthelmintics. Future studies can build on this research by investigating the optimal dosage and delivery methods for these nanoparticles, as well as their efficacy in vivo and in field trials.

## Conclusion

This study investigated the antiparasitic properties of AgNPs, SeNPs, and PPE against *H. contortus* in vitro and determined the LD50 for these compounds. The results demonstrate that AgNPs, SeNPs, and PPE have potent anthelmintic activity against *H. contortus* at different stages of its life cycle. This study is the first to characterize the antiparasitic capabilities of these compounds in this host. These findings suggest that AgNPs, SeNPs, and PPE hold potential as alternative approaches against *H. contortus*, which is crucial considering the escalating prevalence of anthelmintic resistance and the necessity for sustainable parasite control strategies.

## Data Availability

Not applicable and all data are presented in the manuscript.
